# Endoscopic membranectomy for congenital duodenal stenosis in an adult

**DOI:** 10.1055/a-2262-8128

**Published:** 2024-03-08

**Authors:** Ran Chen, Shiya Hong, Zhi Ni, Qingyong Zhang, Xiaoqing Huang, Lan Lin, Rongchun Zhang

**Affiliations:** 1 Department of Gastroenterology, Xiamen Humanity Hospital, Fujian Medical University, Xiamen, China

**Fig. 1:**
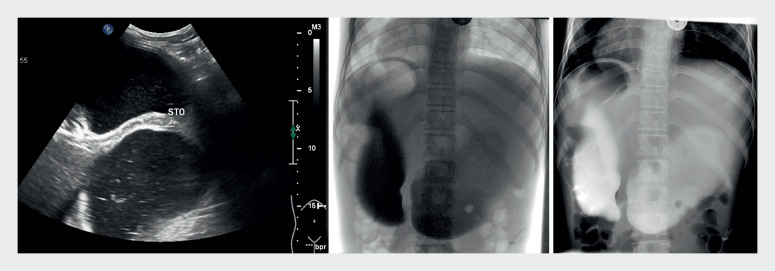
Abdominal ultrasound and X-ray “double-bubble sign.”

**Fig. 2:**
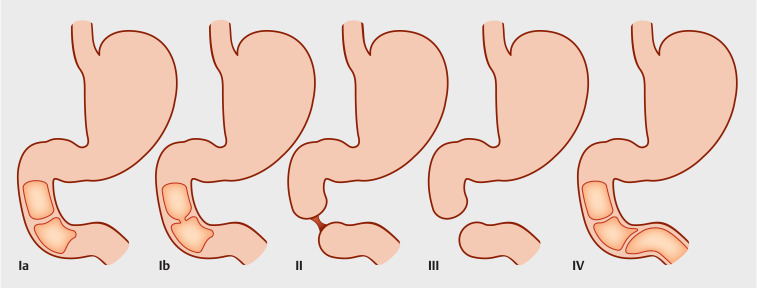
The proposed new classification of congenital duodenal atresia. Type l: duodenal web/membrane. Type la: complete duodenal web/membrane. Type Ib: fenestrated duodenal web/membrane. Type lI: two blind ends of the duodenum with an intact mesentery and separated by a fibrous cord. Type lII: two blind ends of the duodenum with a V-shaped defect in the mesentery. Type lV: multiple duodenal atresias/webs.

A 17-year-old girl with Down syndrome (body mass index 15kg/m ^2^ ) was admitted to our department because of recurrent bilious vomiting since birth. Double-bubble sign on ultrasound and upper gastrointestinal imaging ( Fig. 1 ) indicated obvious dilation of the duodenum and stomach. Congenital stricture of the duodenum is mainly classified into four types ( Fig. 2 ) 1 . Gastroscopy confirmed a membranous duodenal stenosis (Type Ib), with an opening of approximately 1 mm in diameter, and the duodenal papilla was located directly above the diaphragm. We performed membrane radial incision ( Video 1 ).

Endoscopic membranectomy for congenital duodenal stenosis.Video 1

**Fig. 3:**
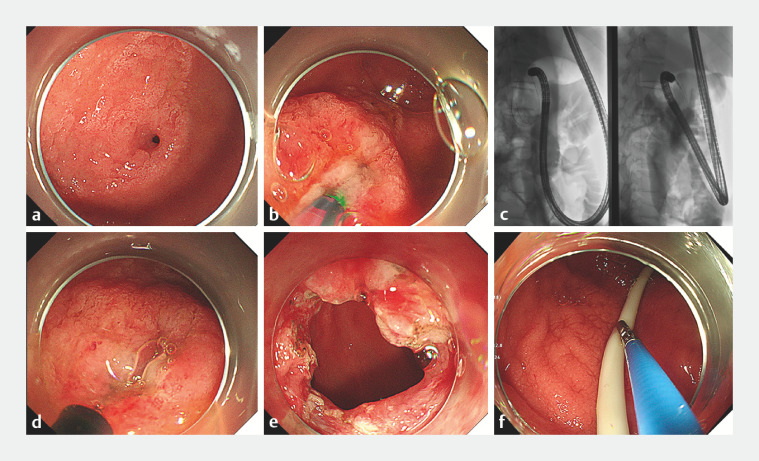
Endoscopic images. **a** Congenital duodenal stenosis. **b** A guidewire probes the enteric cavity. **c** The dilated balloon is pulled back to measure the stenosis thickness. **d** The insulation-tipped knife is used to make a radial incision. **e** An endoscope with outer diameter up to 12 mm is able to pass through the stenosis. **f** The jejunal feeding tube and decompression gastric tube are placed.

**Fig. 4:**
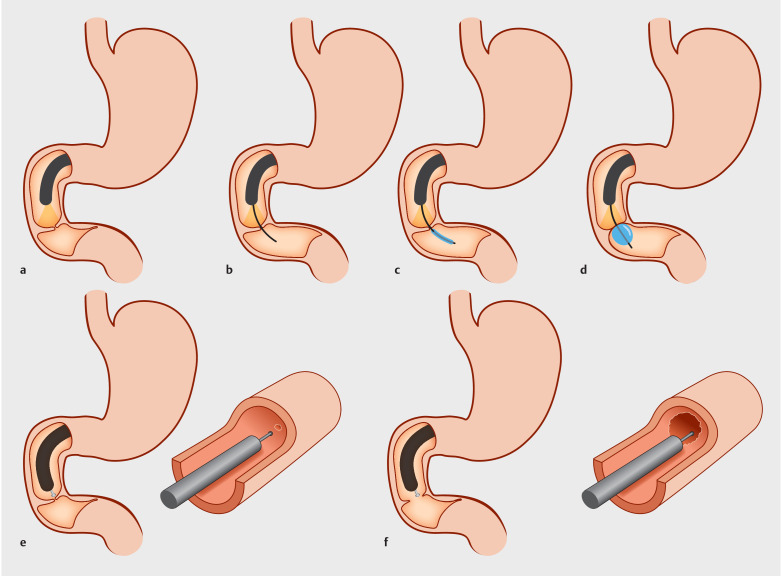
**a** Gastroscopy confirms membranous stenosis. **b** A guidewire probes the intestinal cavity. **c** The balloon follows the guidewire through the stenosis. **d** The dilated balloon is pulled back to measure the stenosis thickness. **e** The insulation-tipped knife is used to make a radial incision, avoiding the duodenal papilla. **f** The 12-mm-diameter endoscope passes through the stenosis.

Membranous stenosis of the descending duodenum was confirmed by gastroscopy ( Fig. 3 **a** , Fig. 4 **a** ). A guidewire was used to probe the enteric cavity ( Fig. 3 **b** , Fig. 4 **b** ), and the dilated balloon was then pulled back to measure the stenosis thickness, which was <1 cm ( Fig. 3 **c** , Fig. 4 **c,d** ). Guided by the guidewire, an insulation-tipped knife was used to make a radial incision, avoiding the duodenal papilla ( Fig. 3 **d** , Fig. 4 **e** ). An endoscope with an outer diameter of ≤12 mm could then pass through the stenosis ( Fig. 3 **e** , Fig. 4 **f** ). After the incision, the wound was treated with thermocoagulation forceps to stop the bleeding, and a nasojejunal tube was inserted through the opening ( Fig. 3 **f** ).

The duodenal incidence of congenital stenosis in newborns is approximately 1.2/10 000 2 , 30%–50% of which are associated with Down syndrome 3 . Endoscopic treatments are still at an exploratory stage. At present, the main treatment methods are radicotomy and balloon dilation 4 . Given that the duodenal papilla of this patient was located directly above the diaphragm, and considering that the radial force exerted by endoscopic balloon dilation on the stenosis is uncontrollable during the expansion process, the muscle layer is likely to be damaged, and there may be a high risk of perforation and duodenal papilla injury. In contrast, duodenal diaphragmatic incision 5 has a controllable and targeted direction for treating stenosis, which can reduce the risk of perforation.

Endoscopy_UCTN_Code_TTT_1AO_2AN
